# Chromosome 8q23.3, 10p14 and 11q23.1 variants modify colorectal cancer risk in Lynch syndrome – a combined analysis of the Australian, Dutch and Polish Lynch syndrome cohorts

**DOI:** 10.1186/1897-4287-10-S2-A32

**Published:** 2012-04-12

**Authors:** Bente A Talseth-Palmer, Juul T Wijnen, Ingvild S Brenne, Shantie Jagmohan-Changur, Katie A Ashton, Carli M Tops, Tiffany-Jane Evans, Mary McPhillips, Claire Groombridge, Janina Suchy, Grzegorz Kurzawski, Allan Spigelman, Pål Møller, Hans M Morreau, Tom Van Wezel, Jan Lubinski, Hans FA Vasen, Rodney J Scott

**Affiliations:** 1 School of Biomedical Sciences and Pharmacy, University of Newcastle, Australia; 2 Hunter Medical Research Institute, John Hunter Hospital, Newcastle, Australia; 3 Center of Human and Clinical Genetics, Leiden University Medical Centre, Leiden, the Netherlands; 4 Department of Pharmacology, The Institute of Pharmacy, Faculty of Medicine, University of Tromsø, Norway; 5 Hunter Area Pathology Service, Hunter New England Area Health, Newcastle, Australia; 6 Hunter Family Cancer Service, Hunter New England Area Health, Newcastle, Australia; 7 International Hereditary Cancer Center, Department of Genetics and Pathology, Pomeranian Academy of Medicine, Szczecin, Poland; 8 The Dutch Cancer Genetics Group, the Netherlands; 9 University of NSW, St Vincent’s Hospital Clinical School, Sydney, Australia; 10 Section for Inherited Cancer, Department of Medical Genetics, Rikshospitalet-Radiumhospitalet Medical Center, Oslo, Norway; 11 Department of Pathology, Leiden University Medical Centre, Leiden, the Netherlands; 12 Dutch Foundation for the Detection of Hereditary Tumours, Leiden, the Netherlands

## Background

For a decade researchers have been searching for modifier genes in individuals with a molecular diagnosis of Lynch syndrome but the task has proven difficult as discordant results seem to be the rule rather than the exception. Recently, two colorectal cancer (CRC) susceptibility loci have been found to be significantly associated with an increased risk of CRC in Dutch Lynch syndrome patients irrespective of which gene was mutated. In a combined study of CRC risk in Australian and Polish Lynch syndrome patients only *MLH1* mutation carriers were found to be at increased risk of disease. A combined analysis of the three datasets was performed to better define this association.

## Methods

The three populations combined totalled 1359 individuals from 425 families with a molecular diagnosis of Lynch syndrome. To date, this represents the largest Lynch syndrome cohort examined for modifier genes. Seven SNPs, from 6 different CRC susceptibility loci, were genotyped by both research groups and the data analysed collectively.

## Results

Individuals with *MLH1* mutations harbouring the CC (variant) genotype of SNP rs3802842 are at increased risk of CRC (HR=2.77, p<0.001) and develop CRC on average 11 years earlier than individuals with the AA (wild type) genotype. All females (*MLH1*, *MSH2* and *MSH6* mutation carriers) carrying the CC genotype of SNP rs3802842 are at increased risk of CRC (HR=2.16, p=0.005), while female MLH1 mutation carriers are at highest risk (HR=3.88, p<0.001).

To investigate whether a cluster of risk alleles increases the risk of CRC, SNP rs3802842 was combined with the other six SNPs additively. MLH1 mutation carriers harbouring 3 risk alleles for SNP combination; rs3802842 (11q23.1) + rs16892766 (8q23.3) display an increased risk of CRC (HR=5.67, p=0.001) and an immense difference in the age of diagnosis of CRC of 28 years is observed compared to individuals with 0 risk alleles. While SNP combination; rs3802842 + rs10795668 (10p14) displays an increased risk of CRC for all females harbouring 4 risk alleles (HR=5.52, p=0.003).

## Conclusion

These results confirm the role of modifier genes in HNPCC. We recommend that Lynch syndrome patients with *MLH1* mutations and all Lynch syndrome females are genotyped for two SNPs in each group so that a personalised risk assessment and tailored surveillance program can be offered to patients at increased risk of CRC and therefore likely to develop their CRCs at much younger ages than the average age of disease onset.

